# Analysis of Potential Risk Factors for Multidrug-Resistance at a Burn Unit

**DOI:** 10.3390/ebj4010002

**Published:** 2023-01-11

**Authors:** Luís Cabral, Leonor Rodrigues, Ana H. Tavares, Gonçalo Tomé, Marisa Caetano, Catarina Chaves, Vera Afreixo

**Affiliations:** 1 Department of Plastic Surgery and Burns Unit, Coimbra University Hospital Centre (CHUC), 3000-075 Coimbra, Portugal; 2 Department of Mathematics, CIDMA—Center for Research and Development in Mathematics and Applications, University of Aveiro, 3810-193 Aveiro, Portugal; 3 Center for Research and Development in Mathematics and Applications—School of Technology and Management (ESTGA), 3750-127 Águeda, Portugal; 4 Pharmacy Department, Coimbra University Hospital Centre (CHUC), 3000-561 Coimbra, Portugal; 5 Clinical Pathology Department, Coimbra University Hospital Centre (CHUC), 3000-561 Coimbra, Portugal

**Keywords:** multidrug-resistant bacteria, infection, risk factors, burns

## Abstract

Background: Infections by multidrug-resistant (MDR) microorganisms are associated with increased morbidity and mortality in burn patients. This study aimed to analyze the evolution of MDR bacteria over a five-year period at Coimbra Burns Unit (CBU) in Portugal, seeking to assess the possible associations of specific bacteria with presumed risk factors. Methods: The data obtained consisted of identified bacteria present in any microbiological sample from each patient (including blood, central venous catheter, urine, tracheal aspirate and/or wound exudate). Univariate models and a multivariate model were constructed for each of the MDR bacteria species that infected at least 50 patients or that had five or more MDR strains. Statistical hypothesis tests with a p-value less than 0.05 were considered significant. Results: Of a total of 341 samples obtained, 107 were MDR, corresponding to 10 species. Globally, there was no significant variation in MDR bacteria frequency over the period under analysis. Some risk factors and/or trends were identified for some species, but none was linked to all of them. Conclusions: The risks for the development of MDR in bacteria in burn patients are multifactorial, mainly linked to longer hospital stays, the use of invasive devices and inadequate antimicrobial treatment. However, the influence of these risks regarding specific bacterial species is not straightforward and may rely on individual characteristics, type of treatment and/or local prevalent flora. Due to the severity of multidrug-resistant infections, continued microbiological surveillance with the aid of rapid diagnostic tests and prompt institution of appropriate antimicrobial therapy are crucial to improving outcomes for burn patients.

## 1. Introduction

Burns are a common cause of morbidity and mortality worldwide [1], accounting for approximately 180,000 deaths each year [2], mostly in low-income countries [3]. In extensive burns, patients need to be admitted to burn units due to the large homeostatic changes caused by leaks from bare areas and associated pain. These patients are also at high risk of infection [4] due to various causes, including intrinsic factors (loss of the skin barrier, trauma-induced immunosuppression, presence of necrotic tissues, gut microbial translocation, reduced airway clearance, etc.) and extrinsic factors (immobilization, nosocomial flora, mechanical ventilation, intravenous access, urinary catheters, etc.) [5,6].

Bacterial colonization, that is, the simple presence of microorganisms, pathogenic or not, on the surface of the burn, without deeper tissue invasion or clinical signs and symptoms, is often followed by infection when microorganisms invade and multiply in the underlying tissues, causing signs and symptoms. For different biological samples, it is important to mention the type of collection and the type of sample, as it directly implies the interpretation of infection versus colonization. When the microbial invasion reaches the bloodstream, the infection becomes systemic and an inflammatory response can reach such a level that deleterious pathophysiological changes occur in the host, characterizing a scenario of sepsis [7,8]. In fact, sepsis is now the main cause of death in burn patients, reaching a rate that varies from 6% to 65% [9,10,11,12]. Physicians should always be aware of signs of sepsis (tachypnea, hypotension, mental alteration, unexplained hyperglycemia) and biomarkers can be used to aid in its diagnosis [13]. If there is a strong clinical suspicion of systemic infection, particularly if also supported by laboratory analysis, empiric broad-spectrum antimicrobials should be administered without delay, with de-escalation when susceptibility testing is available [14]. Unfortunately, mainly due to the misuse of antimicrobials in recent decades, nosocomial bacteria are often resistant to one or more antimicrobial groups, which implies that the local pattern of microbial resistance must be taken into account when choosing antimicrobials [15,16]. On the other hand, in the absence of suspected sepsis, the administration of antimicrobials is not only superfluous, but also has potentially deleterious effects, promoting the development of microbial resistance. In other words, pure prophylactic administration of antimicrobials should be avoided, even in patients with extensive burns.

Multidrug resistance (MDR) is defined as the non-susceptibility of a microorganism to at least one agent in three or more antimicrobial classes. Extensive drug resistance (XDR) is a particular type of multidrug resistance defined as non-susceptibility to at least one agent in all but two or fewer antimicrobial classes (i.e., bacterial isolates are only susceptible to agents of one or two groups), while the expression “pandrug resistance” (PDR) is used to refer non-susceptibility to all agents of all classes (i.e., no agent tested is effective against the microorganism) [17]. Due to the greater severity of infection by multidrug-resistant microorganisms, strict control of the infection, microbiological monitoring, as well as the most appropriate treatment, including the rational use of antimicrobials and rapid debridement and coverage of burned areas, are extremely important. Prompt discontinuation of superfluous antibiotic therapy, as well as removal of all invasive devices as soon as possible, should also be ensured to reduce the risks of developing microbial resistance.

In this study, the evolution of bacterial multidrug resistance was statistically analyzed over five years at the Coimbra Burns Unit (CBU) in Portugal, aiming to assess the possible associations between specific bacteria and the presumed risk factors.

## 2. Materials and Methods

In order to carry out the present study, a database from the Coimbra Burns Unit (CBU), a Department of Centro Hospitalar e Universitário de Coimbra—CHUC, in Portugal, was used, focusing on the five-year period between 1 January 2016 and 31 December 2020. As this work is a retrospective observational study of patients’ health records from a duly anonymized dataset, the CHUC Ethics Committee, in accordance with the Declaration of Helsinki and the Ethical Guidelines International Council for International Organizations of Medical Sciences (CIOMS) waived the need for informed consent. Each patient admitted to CBU during this period was taken as a sampling unit, and when more than one admission was found, only the information regarding the first hospitalization was considered.

The data obtained consisted of the identification of bacteria present in any microbiological sample of each patient (including blood, central venous catheter, urine, tracheal aspirate and/or wound exudate) and the respective antibiotic sensitivity tests (AST). Bacteria species resistant to at least one antibiotic from three different antibiotic classes were considered to be multidrug-resistant. Bacterial species resistant to at least one antibiotic from three different classes of antibiotics were considered multiresistant. For this study, all species of bacteria that infected at least 50 patients were included, as well as those that, despite infecting a smaller number of patients, had at least five MDR strains registered.

Qualitative variables were described as absolute and relative frequencies and quantitative variables as medians, minimum and maximum values. The comparison of each of the variables was carried out over the years. Pearson’s chi-square test was used for qualitative variables and the Kruskal–Wallis rank sum test was used for quantitative variables. Binary logistic regression models were also used for the statistical study. Univariate models and a multivariate model were constructed for each bacterial species that met the study inclusion criteria. MDR was used as a dependent variable and the following parameters were considered as predictor variables: year, age, sex, TBSA, burn degree, length of stay, central venous catheter insertion, mechanical ventilation, length of mechanical ventilation and airway injury. To integrate independent variables in the multivariate model, the selection criteria was the *p*-value of each variable being less than 0.25 in the univariate model. The predictor variable year was always included in the multivariate models. To analyze multicollinearity problems, the variance inflation factor (VIF) was used. A model was considered to have this problem if the VIF of at least one independent variable was greater than 3. The independent variables “mechanical ventilation” and “airway injury” are potentially correlated. Thus, if a model had multicollinearity problems and these variables appeared together in the model, the variable mechanical ventilation was excluded. For statistical purposes, all patients subjected to mechanical ventilation were considered as having air injury, no matter the cause of it. If this problem was due to other variables, the variables with higher VIF were gradually excluded until the VIF of all the variables remaining in the model was up to 3.

Statistical hypothesis tests with a *p*-value less than 0.05 were considered significant. All statistical analyses were performed using R^®^ software (version 4.1.0), R Foundation for Statistical Computing, Vienna, Austria.

## 3. Results

The study sample was composed of 341 patients (176 men—51.6% and 165 women—48.4%) admitted to CBU during the five-year period between January 1st, 2016 and December 31st, 2020. As mentioned, only bacteria species that infected at least 50 patients or that had 5 or more MDR strains were included (Table 1).

The patients’ ages varied from 18 years to 99 years old with a median of 67 years. The most common causes of burns were flames (206 patients—60.4%) and hot liquids (96 patients—28.2%). The TBSA varied from 0.5% to 95% with a median of 10%. A total of 72 patients (21.1%) had 2nd degree burns, 222 (65.1%) had 2nd and 3rd degree burns, and 47 (13.8%) had 3rd degree burns. The days of hospitalization ranged from 2 to 160 with a median of 20 days. Central venous catheter insertion was performed in 126 patients (37.0%), while 116 patients (34.0%) required mechanical ventilation. The duration of mechanical ventilation ranged from 1 day to 159 days with a median of 15 days. At least one multidrug-resistant microorganism was isolated from 107 patients (31.4%). The sample mortality rate was 12.6%, with 43 patients deceased. With the exception of central venous catheter insertion, there was homogeneity over the years (*p* < 0.001) for the studied variables. These results are shown in Table 2.

**Figure 1:**
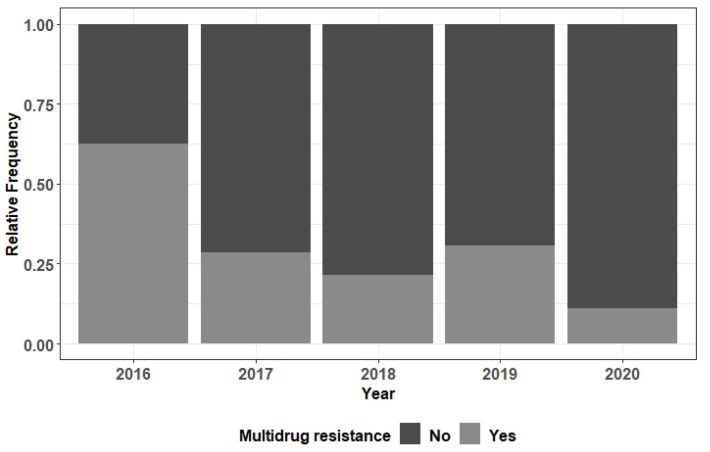
Barplot of the multidrug resistance relative frequency in function of year—*Serratia marcescens*.

Overall, there was no significant variation in the frequency of MDR bacteria over the period under review (2016 to 2020). However, in relation to *Serratia marcescens*, and considering the univariate model that contains only the variable “year”, the risk of developing MDR was significantly lower in patients from 2020 when compared to the ones from 2016 (OR = 0.08, *p* = 0.044) (Figure 1); the significance of these differences was lost when the model was adjusted with other predictive variables (gender, central venous catheter insertion and duration of mechanical ventilation).

**Figure 2:**
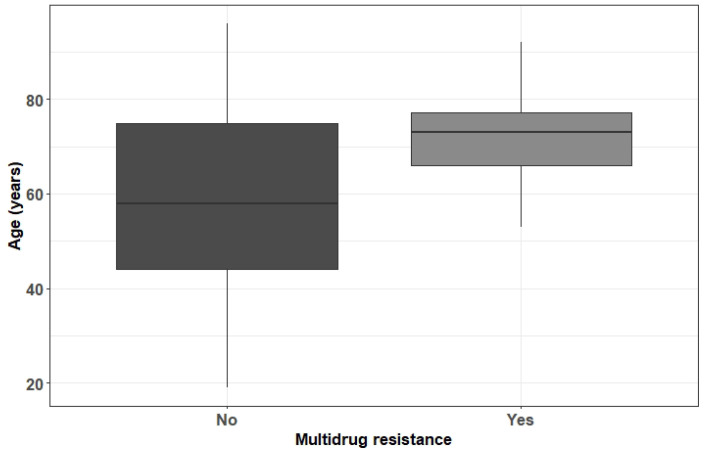
Boxplot of the age in function of multidrug resistance—*Staphylococcus aureus*.

For *Staphylococcus aureus*, it was found that increasing age is a risk factor for the development of MDR (OR = 1.05, *p* = 0.028) (Figure 2).

Mechanical ventilation and airway injury were also risk factors in this sample, except for *Klebsiella pneumoniae*, *Proteus mirabilis* and *Acinetobacter baumannii*.

There was a general trend towards an increased risk for the development of MDR in patients with central catheter venous insertion, except for *Proteus mirabilis*. No apparent trends were found for age, sex and TBSA. For some species, the increase in the magnitude of these last three variables led to a risk effect, but in other cases, a protective effect was found, and finally, in some cases, a null effect was verified. However, in both cases (protective and risk effect) the OR values were all very close to unit (null effect). In other cases, the effect size of the predictor variable burn degree was not possible to estimate (convergence problems), which made it difficult to achieve a consistent conclusion about this variable. The observation of these trends did not imply the existence of significant results; the trends were analyzed even in its absence.

Microbial resistance rates for all of the microorganisms at CBU have been consistently below the average of CHUC along the sample period, and most probably is related to sustained infection control measures and antimicrobial stewardship. Following the general tendency of the hospital, beta-lactams and quinolones were the antimicrobial classes which presented the most resistance at the sensibility tests.

The summary of the results of the univariate and multivariate models for the bacteria species considered in the study is represented in Table S1.

## 4. Discussion

Infection remains the leading cause of complications and fatalities in burn patients, requiring prompt and adequate antimicrobial therapy to improve the outcome. Unfortunately, the development of multidrug resistance by various pathogens in recent decades, particularly for *Staphylococcus aureus* and Gram-negative bacteria, seriously challenging its treatment and increasing mortality rates, has become a widespread dangerous reality [18,19,20]. The problem affects all countries in the world, but it is even more serious in low-income countries where infection control is hampered by socioeconomic factors and access to new antimicrobials, especially developed for multidrug-resistant patients, is quite difficult.

Over time, changes were noted not only in the temporal evolution of microbial colonization in burned areas, which naturally ends up being reflected in the etiology of septic outbreaks, but also in the frequency of different pathogens and their resistance patterns. These alterations, despite having some regional and national component, can be described as being mostly local, varying the parameters from burn unit to burn unit [21]. It is therefore extremely important to consistently study both the microbial flora prevalent in each of the facilities and the evolution of its resistance in order to optimize the choice of the most effective therapeutic agents and reduce the selective pressure on the microorganisms.

Typically, on hospital admission, burns are colonized by Gram-positive bacteria from adjacent unburned areas of skin [22]. After the end of the first week of hospitalization, however, the wound was already colonized mainly by Gram-negative bacteria, originating from the patient’s digestive and respiratory tracts or from cross-infection by health professionals [23]. Finally, more often after the third week, opportunistic fungal or viral colonization may occur after prolonged antibiotic therapy and/or clinical degradation [24]. When colonizing microorganisms develop multidrug resistance, thus increasing their survival against current antimicrobials, there is naturally a greater likelihood of invasive skin infections and sepsis [25]. Gram-negative bacteria are inherently resistant to many antibiotics and are also more likely to develop multidrug resistance than Gram-positive bacteria, potentially being more virulent, leading to infections with higher mortality rates and higher treatment costs [26,27]. In the present study, the frequency of different MDR microorganisms was similar to that found in other series, including burned and non-burned patients [28,29].

With a higher risk of infection due to multifactorial causes, patients with severe burns are also subject to some factors predisposing them to the development of multidrug resistance, in addition to inadequate and/or prolonged antimicrobial therapy. The literature confirms that long hospital stays, prolonged mechanical ventilation and/or the use of central venous catheters and other invasive devices, as well as previous courses of broad-spectrum antimicrobials are some of these factors, but they do not have the same importance according to different microbial species [30,31,32], which was also found in the present study. Strict infection control protocols, contact isolation of patients infected with MDR microorganisms, timely surgery and the implementation of an antimicrobial management program according to the local bacterial flora are consensually recommended strategies for the prevention and management of MDR bacterial infections [33]. The prophylactic administration of antibiotics remains a controversial topic. Although most authors do not recommend it due to the scarce evidence of its effectiveness and the increased risk of selection of MDR microorganisms [34,35], others mention a positive effect in ventilated patients, with a reduction in mortality at 28 days, however absent in non-ventilated ones [36]. Meanwhile, a large multicenter, prospective, randomized, double-blind, placebo-controlled trial is ongoing, with the aim of recruiting 506 adult burn patients, having between 5 and 40% TBSA and requiring at least a deep burn excision graft surgery, to assess the validity of such a strategy [37].

One of the strengths of this study is that it includes multiple MDR microorganisms, rather than limiting analysis to one or a select few. On the other hand, either the microbiological sample collection, surgical and antimicrobial treatment (when necessary) and statistical analysis were performed according to the same protocols during a period of five years. The study obviously has the limitation of being retrospective and performed with patients from a single center. Furthermore, the reduced sample size for some species of bacteria may have influenced the results, leading to greater variability and, consequently, to some degree of inconsistency in the results. In addition, it was not possible to perform a sub-analysis according to the different sample collection sites, which could refine the results.

## 5. Conclusions

The risks for the development of multidrug resistance in bacteria colonizing and/or infecting burn patients increases are multifactorial, mostly linked to longer length of stay, use of invasive devices and inadequate antimicrobial treatment. However, according to the results of this study, the influence of such risks regarding specific bacterial species is not straightforward and may rely on individual characteristics, type of treatment and/or local prevalent flora. Due to the much greater morbidity and mortality linked to MDR infections, a permanent microbiological surveillance, with the help of quick diagnostic tests [38,39,40], and the immediate institution of an adequate antimicrobial therapy are crucial to improve the outcomes. It has been proved that as soon as pathogen identification and sensitivities are known, antibiotic de-escalation strategies lead to a reduction of the development of multidrug therefore being highly recommended [41,42,43].

Gathering a more substantial sample for statistical analysis, a multicentric study predictably could give stronger insights about the potential influence of each specific risk of multidrug resistance for different bacteria species or confirm the absence of effect.

## Figures and Tables

**Table 1:** Prevalence of bacterial species and MDR strains (2016–2020).

Bacteria Species	Number of Patients	MDR ^a^ Strains
*Staphylococcus aureus*	152	10
*Enterococcus faecalis*	76	3
*Pseudomonas aeruginosa*	73	29
*Escherichia coli*	63	13
*Klebsiella pneumoniae*	60	24
*Serratia marcescens*	51	15
*Proteus mirabilis*	49	13
*Enterobacter cloacae*	46	7
*Enterococcus faecium*	13	9
*Acinetobacter baumannii*	12	6

^a^ Multidrug resistance.

**Table 2:** Characteristics of the patients.

Characteristic	All	2016	2017	2018	2019	2020	*p*
***n***	341	65	56	83	75	62	
**Age** (years)	67(18–99)	70(20–92)	59(20–96)	65(24–96)	74(18–99)	67(19–90)	0.067 ^c^
**Sex**							
Female	165(48.4)	38(58.5)	20(35.7)	41(49.4)	41(54.7)	25(40.3)	0.059 ^d^
Male	176(51.6)	27(41.5)	36(64.3)	42(50.6)	34(45.3)	37(59.7)	
**TBSA** ^a^ (%)	10(0.5–95)	13(1–83)	17.5(0.5–72)	8.0(1–62)	6.5(1–95)	10.5 (1–95)	0.006 ^c^
**Burn degree**							
2nd	72(21.1)	14(21.5)	17(30.4)	18(21.7)	13(17.3)	10(16.1)	0.709 ^d^
2nd and 3rd	222(65.1)	41(63.1)	31(55.4)	53(63.9)	53(70.7)	44(71.0)	
3rd	47(13.8)	10(15.4)	8(14.3)	12(14.5)	9(12.0)	8(12.9)	
**Length of stay** (days)	20(2–160)	29(2–152)	19.5(2–160)	18(2–129)	17(3–78)	22(4–107)	0.051 ^c^
**Central venous** **catheter**							
Yes	126(37.0)	39(60.0)	32(57.1)	27(32.5)	22(29.3)	6(9.7)	**<0.001** ^d,e^
No	215(63.0)	26(40.0)	24(42.9)	56(67.5)	53(70.7)	56(90.3)	
**Mechanical** **ventilation**							
Yes	116(34.0)	27(41.5)	22(39.3)	22(26.5)	21(28.0)	24(38.7)	0.182 ^d^
No	225(66.0)	38(58.5)	34(60.7)	61(73.5)	54(72.0)	38(61.3)	
**Length of** **mechanical** **ventilation** (days)	15(1–159)	22(3–88)	21(2–159)	11.5(2–67)	10(1–48)	16(2–58)	0.068 ^c^
**MDR** ^b^							
Yes	107(31.4)	23(35.4)	18(32.1)	21(25.3)	26(34.7)	19(30.6)	0.678 ^d^
No	234(68.6)	42(64.6)	38(67.9)	62(74.7)	49(65.3)	43(69.4)	
**Death**							
Yes	43(12.6)	5(7.7)	6(10.7)	10(12.0)	13(17.3)	9(14.5)	0.500 ^d^
No	298(87.4)	60(92.3)	50(89.3)	73(88.0)	62(82.7)	53(85.5)	

^a^ Total body surface area; ^b^ Multidrug resistance; ^c^ Kruskal–Wallis rank sum test; ^d^ Pearson’s Chi-squared test; ^e^ Significant, *p* < 0.05.

## Data Availability

Data supporting the reported results can be found at The Clinical Pathology Department of Coimbra University Hospital Center.

## Supplementary Materials

The following supporting information can be downloaded at: https://www.mdpi.com/article/10.3390/ebj4010002/s1, Table S1: Univariate and multivariate logistic regression models para all bacteria species in analysis.
