# Supplementary material for: Analysis of Potential Risk Factors for Multidrug-Resistance at a Burn Unit
Source: Eur Burn J. 2023 Jan 11;4(1):9–17. doi: 10.3390/ebj4010002 (PMC11571875; doi:10.3390/ebj4010002)
Supplement: Supplementary file 1 [file ebj-04-00002-s001.zip › ebj-2051033-supplementary.pdf]

Table S1. Univariate and multivariate logistic regression models para all bacteria species in analysis

Bacteria species	Model	Year (ref. 2016)								Age (years)		Sex (Male)		TBSA (%)		Burn degree (ref. 2nd)				Length of stay (Days)		Central venous catheter (Yes)		Mechanical ventilation (Yes)		Length of mechanical ventilation (Days)		Airway injury (Yes)	
		2017		2018		2019		2020								2nd + 3rd		3rd											
		OR[a]	CI$_{95\%}$[b]	OR[a]	CI$_{95\%}$[b]	OR[a]	CI$_{95\%}$[b]	OR[a]	CI$_{95\%}$[b]	OR[a]	CI$_{95\%}$[b]	OR[a]	CI$_{95\%}$[b]	OR[a]	CI$_{95\%}$[b]	OR[a]	CI$_{95\%}$[b]	OR[a]	CI$_{95\%}$[b]	OR[a]	CI$_{95\%}$[b]	OR[a]	CI$_{95\%}$[b]	OR[a]	CI$_{95\%}$[b]	OR[a]	CI$_{95\%}$	OR[a]	CI$_{95\%}$[b]
Staphylococcus aureus	Uni.[c]	1.93	0.18, 42.9	3.41	0.47, 68.8	2.64	0.32, 54.9	[f]	–	1.05	1.01, 1.10	0.84	0.23, 3.16	1.01	0.96, 1.04	0.53	0.13, 2.66	0.36	0.02, 3.06	1.01	0.99, 1.03	2.16	0.57, 8.11	1.31	0.32, 4.79	0.98	0.89, 1.03	1.39	0.34, 5.11
		p = 0.599		p = 0.284		p = 0.412		–		**p = 0.034**[e]		p = 0.796		p = 0.773		p = 0.395		p = 0.394		p = 0.213		p = 0.243		p = 0.690		p = 0.528		p = 0.622	
	Multi.[d]	2.89	0.24, 67.8	5.15	0.64, 111	3.74	0.41, 83.2	[f]	–	1.05	1.01, 1.10									1.01	0.99, 1.04	1.73	0.29, 8.92						
		p = 0.413		p = 0.172		p = 0.285		–		**p = 0.028**[e]										p = 0.295		p = 0.520							
Enterococcus faecalis	Uni.[c]	3.17	0.27, 72.6	[f]	–	[f]	–	[f]	–	1.03	0.95, 1.13	1.84	0.17, 40.7	1.02	0.95, 1.07	[f]	–	[f]	–	1.02	0.99, 1.05	1.48	0.14, 32.6	2.17	0.20, 47.9	1.02	0.98, 1.07	2.71	0.25, 59.9
		p = 0.367		–		–		–		p = 0.547		p = 0.624		p = 0.523		–		–		p = 0.216		p = 0.755		p= 0.534		p = 0.262		p = 0.424	
	Multi.[d]	3.51	0.30, 83.4	[f]	–	[f]	–	[f]	–											1.01	0.98, 1.04								
		p = 0.333		–		–		–												p = 0.485									
Pseudomonas aeruginosa	Uni.[c]	0.38	0.06, 1.93	0.31	0.07, 1.28	0.64	0.16, 2.48	0.87	0.17, 4.43	0.99	0.96, 1.01	1.55	0.61, 4.06	1.05	1.02, 1.09	5.54	1.33, 38.0	3.00	0.40, 28.0	1.03	1.01, 1.05	5.07	1.88, 14.8	3.51	1.34, 9.64	1.05	1.02, 1.09	4.25	1.59, 12.0
		p = 0.256		p = 0.113		p = 0.516		p = 0.870		p = 0.332		p = 0.363		**p = 0.005**[e]		**p = 0.036**[e]		p = 0.291		**p = 0.006**[e]		**p = 0.002**[e]		**p = 0.012**[e]		**p = 0.005**[e]		**p = 0.005**[e]	
	Multi.[d]	0.50	0.04, 4.50	0.47	0.07, 2.68	0.96	0.19, 4.78	1.95	0.24, 18.3					1.02	0.97, 1.07	3.07	0.54, 26.0	2.34	0.20, 30.9	1.00	0.97, 1.03	2.31	0.39, 14.1			1.03	0.99, 1.08	0.99	0.18, 4.76
		p = 0.548		p = 0.396		p = 0.959		p = 0.540						p = 0.490		p = 0.235		p = 0.494		p = 0.950		p = 0.351				p = 0.213		p = 0.985	
Escherichia coli	Uni.[c]	3.00	0.36, 64.2	2.45	0.26, 54.5	2.08	0.22, 45.8	3.60	0.28, 89.8	0.99	0.96, 1.02	0.49	0.10, 1.84	1.02	0.98, 1.05	3.87	0.63, 74.9	3.43	0.28, 82.1	1.01	0.99, 1.03	1.61	0.47, 5.68	3.00	0.86, 10.9	1.04	1.00, 1.07	3.69	1.04, 13.7
		p = 0.361		p = 0.469		p = 0.553		p = 0.341		p = 0.600		p = 0.321		p = 0.389		p = 0.220		p = 0.348		p = 0.262		p = 0.446		p = 0.086		**p = 0.032**[e]		**p = 0.044**[e]	
	Multi.[d]	5.98	0.60, 149	4.03	0.32, 108	3.72	0.33, 96.3	3.20	0.21, 87.9							3.46	0.45, 74.5	4.74	0.32, 132							1.03	0.98, 1.08	1.84	0.22, 13.6
		p = 0.172		p = 0.311		p = 0.328		p = 0.412								p = 0.298		p = 0.272								p = 0.310		p = 0.550	
Klebsiella pneumoniae	Uni.[c]	0.35	0.06, 1.81	1.17	0.27, 5.19	0.78	0.17, 3.53	0.58	0.02, 7.68	1.01	0.98, 1.03	0.85	0.30, 2.39	1.03	1.00, 1.07	2.73	0.70, 13.6	0.60	0.03, 6.29	1.00	0.99, 1.02	1.55	0.52, 4.86	0.80	0.28, 2.26	1.01	0.98, 1.03	0.85	0.30, 2.39
		p = 0.223		p = 0.837		p = 0.743		p = 0.689		p = 0.642		p = 0.752		p = 0.051		p = 0.172		p = 0.690		p = 0.653		p = 0.441		p = 0.673		p = 0.513		p = 0.752	
	Multi.[d]	0.26	0.04, 1.60	1.15	0.21, 6.32	1.08	0.19, 6.01	0.50	0.02, 7.55					1.03	1.00, 1.07	1.97	0.43, 10.7	0.49	0.02, 5.95										
		p = 0.160		p = 0.868		p = 0.928		p = 0.624						p = 0.095		p = 0.392		p = 0.597											

[a] Odd Ratio; [b] 95% Confidence Interval; [c] Univariate; [d] Multivariate; [e] Significant, $p < 0.05$; [f] Convergence problems (small sample size)

(Continuation)

Bacteria species	Model	Year (ref. 2016)								Age (years)		Sex (Male)		TBSA (%)		Burn degree (ref. 2nd)				Length of stay (Days)		Central venous catheter (Yes)		Mechanical ventilation (Yes)		Length of mechanical ventilation (Days)		Airway injury (Yes)	
		2017		2018		2019		2020								2nd + 3rd		3rd											
		OR[a]	$CI_{95\%}$[b]	OR[a]	$CI_{95\%}$[b]	OR[a]	$CI_{95\%}$[b]	OR[a]	$CI_{95\%}$[b]	OR[a]	$CI_{95\%}$[b]	OR[a]	$CI_{95\%}$[b]	OR[a]	$CI_{95\%}$[b]	OR[a]	$CI_{95\%}$[b]	OR[a]	$CI_{95\%}$[b]	OR[a]	$CI_{95\%}$[b]	OR[a]	$CI_{95\%}$[b]	OR[a]	$CI_{95\%}$[b]	OR[a]	$CI_{95\%}$	OR[a]	$CI_{95\%}$[b]
Serratia marcescens	Uni.[c]	0.24	0.02, 1.93	0.16	0.02, 1.03	0.27	0.04, 1.62	0.08	0.00, 0.71	1.02	0.99, 1.06	0.38	0.11, 1.36	0.99	0.96, 1.03	0.83	0.19, 4.44	4.67	0.32, 127	1.01	0.99, 1.03	4.14	0.95, 29.1	1.60	0.47, 6.02	1.02	1.00, 1.05	1.79	0.52, 6.73
		p = 0.199		p = 0.064		p = 0.162		**p = 0.044**[e]		p = 0.284		p = 0.135		p = 0.663		p = 0.816		p = 0.273		p = 0.237		p = 0.088		p = 0.464		p = 0.149		p = 0.364	
	Multi.[d]	0.20	0.01, 2.20	0.22	0.03, 1.58	0.49	0.06, 3.64	0.20	0.01, 3.33			0.41	0.08, 1.88									2.56	0.37, 24.7			1.02	0.99, 1.05		
		p = 0.216		p = 0.146		p = 0.491		p = 0.291				p = 0.250										p = 0.364				p = 0.264			
Proteus mirabilis	Uni.[c]	[f]	–	3.43	0.35, 78.6	4.80	0.63, 101	3.43	0.35, 78.6	1.00	0.97, 1.04	1.71	0.46, 6.33	0.99	0.94, 1.03	0.92	0.15, 7.33	[f]	–	1.00	0.97, 1.01	0.63	0.16, 2.24	0.20	0.03, 0.90	0.88	0.68, 0.99	0.25	0.04, 1.13
		–		p = 0.330		p = 0.184		p = 0.330		p = 0.923		p = 0.414		p = 0.615		p = 0.926		–		p = 0.639		p = 0.477		p = 0.057		p = 0.147		p = 0.103	
	Multi.[d]	[f]	–	2.72	0.25, 65.9	4.29	0.51, 93.8	3.20	0.29, 77.7																	0.89	0.62, 1.05	0.75	0.07, 9.21
		–		p = 0.445		p = 0.232		p = 0.376																		p = 0.323		p = 0.811	
Enterobacter cloacae	Uni.[c]	0.33	0.01, 3.27	0.27	0.01, 2.55	0.53	0.02, 5.65	0.33	0.01, 3.27	1.03	0.99, 1.08	0.28	0.04, 1.47	1.02	0.99, 1.06	[f]	–	[f]	–	1.03	1.00, 1.05	5.14	0.78, 102	7.00	1.06, 139	1.01	0.99, 1.04	1.73	0.34, 9.76
		p = 0.383		p = 0.290		p = 0.625		p = 0.383		p = 0.137		p = 0.154		p = 0.124		–		–		**p = 0.028**[e]		p = 0.146		p = 0.084		p = 0.220		p = 0.511	
	Multi.[d]	0.34	0.01, 5.93	0.41	0.02, 6.14	1.00	0.02, 28.0	1.42	0.03, 80.1	1.03	0.97, 1.10	0.53	0.03, 7.00									4.47	0.22, 223	3.99	0.26, 120	1.00	0.97, 1.04		
		p = 0.484		p = 0.531		p = 0.998		p =0.843		p = 0.346		p = 0.622										p = 0.377		p =0.342		p = 0.865			
Enterococcus faecium	Uni.[c]	[f]	–	[f]	–	[f]	–	[f]	–	0.98	0.86, 1.07	2.40	0.20, 59.7	1.00	0.93, 1.07	[f]	–	[f]	–	1.08	1.00, 1.28	6.00	0.51, 155	6.00	0.51, 155	1.03	0.95, 1.15	6.00	0.51, 155
		–		–		–		–		p = 0.691		p = 0.512		p = 0.938		–		–		p = 0.170		p = 0.186		p = 0.186		p = 0.526		p = 0.186	
	Multi.[d]	[f]	–	[f]	–	[f]	–	[f]	–											[f]	–	9.68	0.18, 97125						
		–		–		–		–												–		p = 0.347							
Acinetobacter baumanii	Uni.[c]	[f]	–	[f]	–	[f]	–	[f]	–	0.97	0.90, 1.03	5.00	0.41, 131	0.99	0.94, 1.05	[f]	–	[f]	–	1.01	0.99, 1.04	5.00	0.41, 131	0.40	0.02, 5.77	1.00	0.94, 1.06	1.00	0.08, 12.2
		–		–		–		–		p = 0.302		p = 0.239		p = 0.792		–		–		p =0.409		p = 0.239		p = 0.512		p = 0.976		p > 0.999	
	Multi.[d]	[f]	–	[f]	–	[f]	–	[f]	–			2.92	0.16, 106																
		–		–		–		–				p = 0.481																	

[a] Odd Ratio; [b] 95% Confidence Interval; [c] Univariate; [d] Multivariate; [e] Significant, $p < 0.05$; [f] Convergence problems (small sample size)
